# Analysis of center of mass acceleration and muscle activation in hemiplegic paralysis during quiet standing

**DOI:** 10.1371/journal.pone.0226944

**Published:** 2019-12-20

**Authors:** Wei Wang, Yunling Xiao, Shouwei Yue, Na Wei, Ke Li

**Affiliations:** 1 Laboratory of Motor Control and Rehabilitation, Institute of Biomedical Engineering, School of Control Science and Engineering, Shandong University, Jinan, China; 2 Department of Physical Medicine and Rehabilitation, Qilu Hospital, Shandong University, Jinan, China; 3 Department of Geriatrics, Qilu Hospital, Shandong University, Jinan, China; 4 Suzhou Institute of Shandong University, Suzhou, China; University of L'Aquila, ITALY

## Abstract

Hemiplegic paralysis after stroke may augment postural instability and decrease the balance control ability for standing. The center of mass acceleration (COM_acc_) is considered to be an effective indicator of postural stability for standing balance control. However, it is less studied how the COM_acc_ could be affected by the muscle activities on lower-limbs in post-stroke hemiplegic patients. This study aimed to examine the effects of hemiplegic paralysis in post-stroke individuals on the amplitude and structural variabilities of COM_acc_ and surface electromyography (sEMG) signals during quiet standing. Eleven post-stroke hemiplegic patients and the same number of gender- and age-matched healthy volunteers participated in the experiment. The sEMG signals of tibialis anterior (TA) and lateral gastrocnemius (LG) muscles of the both limbs, and the COM_acc_ in the anterior-posterior direction with and without visual feedback (VF vs. NVF) were recorded simultaneously during quiet standing. The sEMG and COM_acc_ were analyzed using root mean square (RMS) or standard deviation (SD), and a modified detrended fluctuation analysis based on empirical mode decomposition (EMD-DFA). Results showed that the SD and the scale exponent α of EMD-DFA of the COM_acc_ from the patients were significantly higher than the values from the controls under both VF (*p* < 0.01) and NVF (*p* < 0.001) conditions. The RMSs of TA and LG on the non-paretic limbs were significantly higher than those on paretic limbs (*p* < 0.05) for both the patients and controls (*p* < 0.05). The TA of both the paretic and non-paretic limbs of the patients showed augmented α values than the TA of the controls (*p* < 0.05). The α of the TA and LG of non-paretic limbs, and the α of COM_acc_ were significantly increased after removing visual feedback in patients (*p* < 0.05). These results suggested an increased amplitude variability but decreased structural variability of COM_acc_, associated with asymmetric muscle contraction between the paretic and the non-paretic limbs in hemiplegic paralysis, revealing a deficiency in integration of sensorimotor information and a loss of flexibility of postural control due to stroke.

## Introduction

Stable posture control for upright standing is a fundamental motor function for daily activities. Hemiplegic paralysis associated with stroke may augment postural instability, impair balance control for standing, or even increase the risk of falling [1]. A range of tests or scales have been used in clinical evaluation of postural stability in the post-stroke population, such as Berg Balance Scale, the Timed Up and Go Test, Balance Subscale of the Fugl-Meyer Assessment, Postural Assessment Scale for Stroke Patients, Activities-Specific Balance Confidence Scale, and Fullerton Advanced Balance Scale [2]. Even though these tests and scales are relatively fast and do not rely on expensive equipment, the results are subjective and insensitive to the balance-related alterations [2, 3]. The optimal balance evaluation technique should include objective and quantitative measurements that would provide detailed, easily comprehensible, and reliable findings. Evaluating postural stability for balance control during standing may provide quantifications for neuromuscular disorders and develop strategies to predict falling risk of patients during rehabilitation.

A range of techniques, including angular momentums, limb vibrations, trunk force line or movement kinematics, are available in postural stability analysis. Among all these techniques, the acceleration of center of mass (COM_acc_) is relatively easy to be recorded, contains critical information about the interaction and coordination among the joints of whole-body [4, 5]. In addition, the COM_acc_ is highly correlated to the distance between the COP and center of mass (COM) at a given time; and the COP-COM particularly in the anterior-posterior (AP) direction, have been used in examination of aging and hemiplegia [6]. Previous studies on patients suffering stroke and diabetic neuropathy demonstrated that the COP-COM, revealing the synchrony between the COP and COM, provides insights into the understanding and assessing the postural control than using either the COP or the COM separately [7, 8]. The post-stroke patients usually demonstrate an increase in the amplitude variability of COM_acc_ compared to healthy population, indicating declined synchronization between the COP and COM that leads to aggravated postural sway [6, 9]. These studies suggested that the COM_acc_ would be an effective indicator of postural stability for standing balance control particularly for the individuals with stroke.

The activation of calf muscles plays a role in postural control of standing balance [10–12]. The gastrocnemius, specifically the lateral gastrocnemius (LG), prevents the entire body from toppling forward during standing and acts as antagonist of tibialis anterior (TA). A majority of stroke patients show asymmetric muscle activations, manifested by muscle weakness on paretic limbs or excessive contraction on non-paretic limbs during standing, leading to increased postural instability and higher risk of falling [13–15]. However, the surface electromyography (sEMG) of TA showed augmented amplitudes on the paretic limbs of hemiplegic individuals compared to healthy controls during squat exercise [16]. Similar sEMG amplitudes of TA and gastrocnemius muscles have been found between non-paretic limbs of post-stroke patients and the control limbs in healthy individuals during slow locomotion [17].

The amplitude and structural variabilities of sEMG and COM_acc_ signals could reflect the neuromuscular control for standing balance. The amplitude variability of COM_acc_, usually quantified using coefficient of variation or standard deviation (SD), indicates the magnitudes of signals’ fluctuation that may reflect the instability of the motor activity [18]. The amplitude variability of sEMG was estimated by the root mean square (RMS), which reflects the averaged intensity during muscle contraction [19]. The structure of signal variability, usually quantified using dynamical analysis, such as the detrended fluctuation analysis (DFA), indicates the time-dependent changes of the signals’ structure, revealing how a complex physiological system evolves with time [18, 20]. Pathological conditions could disrupt the complex fluctuation pattern and multi-scale correlations of physiological signals [21]. Although the DFA algorithm has been extensively used, this algorithm shows limitations in scaling reliability and accuracy of local trends [22, 23]. An improved DFA algorithm determined local trends by an adaptive data analysis method called empirical mode decomposition (EMD), which showed higher reliability in quantifying trends in heartbeat dynamics and the COP trajectory during quiet standing compared to the traditional algorithm [24, 25]. The scaling exponent (α) closed to 1 revealed high flexible dynamic output and postural stability of neuromuscular system. The α can also reflects an anti-persistence (0 < α < 0.5) and persistence (0.5 < α < 1) patterns, which is highly related the neuromuscular function [26]. For example, previous studies found that the older adults usually exhibit increased α of COP trajectory during standing, reflecting a compromised flexibility in the control strategy and reduced dynamic stability with aging [24].

Maintaining a dynamic balance for standing is a process of sensorimotor integration. Presence or absence of visual feedback has been extensively used in experiment for balance control, especially in terms of COP and COM dynamics [27]. The role of visual conditions in postural control is still controversial. Increased fluctuation but lowered complexity of the dynamic outputs have been found in children, adults or the elderly population by removing visual feedback during standing [5, 24]. But in many others no obvious change was found in the scaling exponent of COM_acc_ in absence of visual feedback during standing [28]. However, consensus probably has been reached that without visual feedback the sensory information would be reduced, rendering a higher requirement for balance control. To compare the postural variability under different visual conditions would be interesting, particularly for the post-stroke patients who have reduced capacity of balance control, because patients may highly rely on their visual feedback to compensate for the lack of motor capacity. In addition, physical examination without visual feedback may facilitate to highlight the pathological characteristics underlying the posture control.

The purpose of this study was to examine the effects of hemiplegic paralysis in post-stroke individuals on the amplitude and structural variability of COM_acc_ and sEMG during quiet standing. Finding out characteristics of the amplitude and structural variability of COM_acc_ and sEMG could facilitate to evaluate the stability and flexibility for postural control, and shed light on the neuromuscular deficiency for standing balance maintenance in post-stroke individuals. The structural variability of COM_acc_ and sEMG was quantified using a modified DFA algorithm based on the EMD analysis. We hypothesized that hemiplegic individuals would show higher amplitude and structural variability of the COM_acc_ in the AP direction and asymmetrical muscle activation variability between the paretic and non-paretic limbs during quiet standing.

## Methods

### Participants

Eleven post-stroke hemiplegic patients and the same number of gender- and age-matched healthy volunteers participated in the experiment. The subjects’ characteristics are exhibited in Table 1. All the patients had been clinically diagnosed with first-ever stroke that occurred within 8 months and had received clinical treatment. The hemiplegic patients who participated in the experiment were all at the fourth stage according to the Brunnstrom stages of stroke recovery. The capacity of neuromuscular function was also examined using Modified Barthel Index (MBI) for each patient (Table 1). All the patients could stand independently for at least 10 min. The individuals with tumor, severe malnutrition, cardiovascular diseases and musculoskeletal injuries of lower extremities were excluded. All the subjects signed an informed consent following the protocols approved by the Institutional Review Board of Shandong University after being notified of the purposes and potential risks of the experiment.

**Table 1:** Subjects’ characteristics.

	Stroke (n = 11)	Healthy (n = 11)
Age (years)	51.55 ± 14.29	51.45 ± 14.08
Sex (n): male/female	7/4	7/4
Time since stroke (months)	3.45 ± 2.11	NA
Paretic side (right/left)	6/5	NA
MBI*	66.82 ± 15.99	NA

Data are presented as mean±standard deviation;

* Modified Barthel Index, a measure of activities of daily living.

### Experimental setup

**Fig 1:**
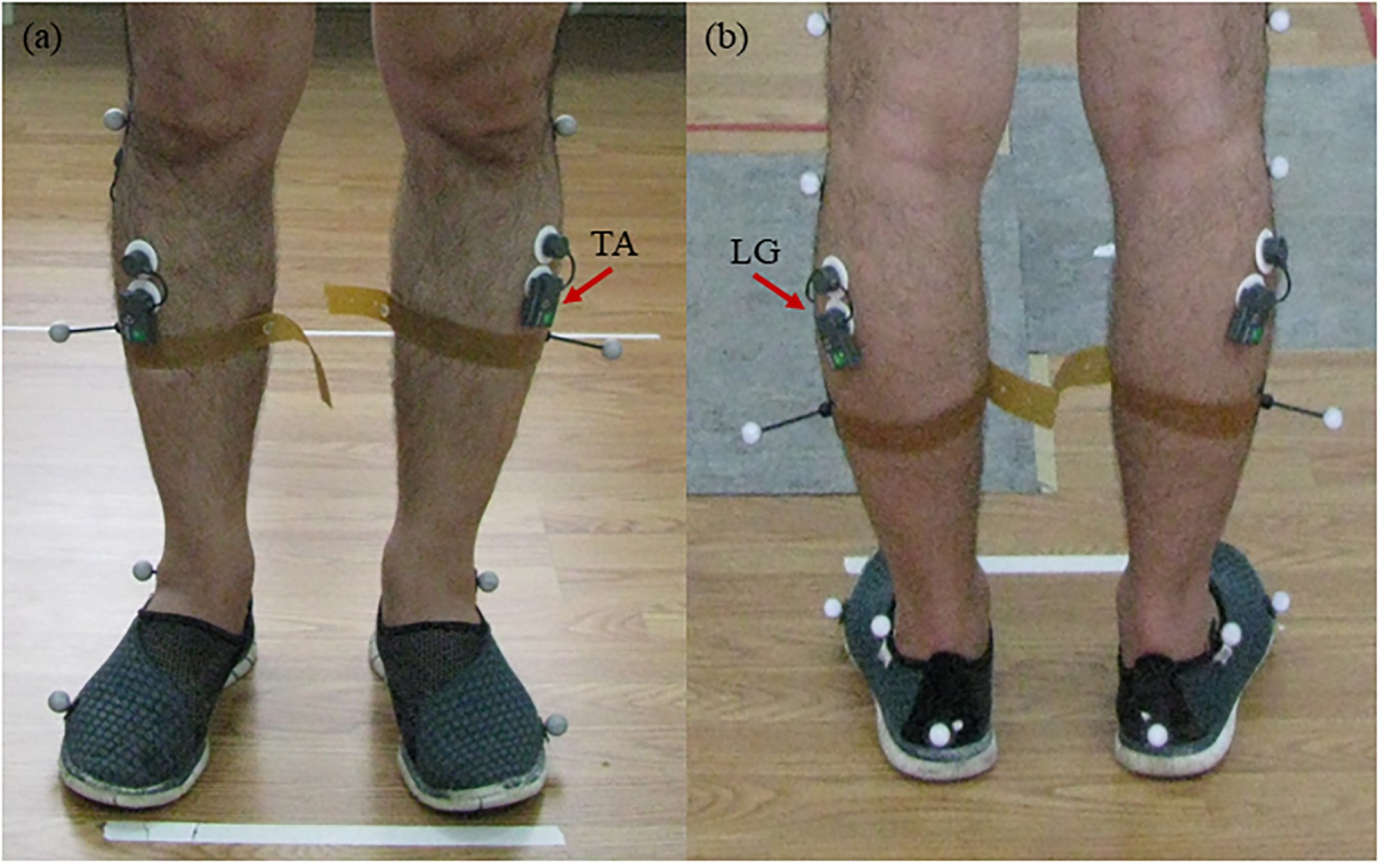
The positions of surface electromyography (sEMG) electrodes. (a) In the front view, (b) in the back view.

A three-dimensional motion acquisition and analysis system (BTS Bioengineering Corp, Italy) was applied in this experiment. The wireless sEMG electrodes (FreeEMG 1000) with 16 bit resolution and 1 kHz acquisition frequency were used to measure the sEMG signals of TA and LG in both lower extremities (Fig 1). The sites of LG muscles were determined at 1/3 of the line between the head of the fibula and the heel. The TA muscles were located at 1/3 on the line between the fibular head and the tip of the medial malleolus. The skin was cleaned by scrub cream and coated with alcohol, before attaching electrodes parallel to the muscle fibers. Two adjacent force plates (INFINI-T, Sensitive area: 60 x 40 cm, Sensitivity/Resolution: 16 bit over selected range), spliced together and embedded horizontally into the ground, recorded force signals synchronously with the sEMG signals. The sampling frequency of sEMG and force signals were 1000 Hz and 400 Hz respectively.

### Test protocol

Subjects were asked to stand upon the centers of force plates, with one foot on each plate, for 1 min. During the first 30 s, subjects were required to maintain their body upright. At the 30^th^ second, a buzzer gave a command, following which the subjects should close their eyes immediately and maintain the exact position for another 30 s (Fig 1). Four trials were performed for each subject with 2-minute interval between trials. Before the formal test subjects were allowed to practice for several times to be familiarized with the protocol, become acclimated to the environment, and relieve tension.

### Data analysis

The ground reaction force of the left and right foot, and the sEMG signals of TA and LG of the both legs were recorded simultaneously. Considering the perturbation in the first and the last 5 s by condition transitions with the VF and NVF during standing, data of the 5–25 s (VF) and 35–55 s (NVF) was used for the following analysis. The COM_acc_ in the AP direction was calculated following Newton’s second law. It was defined as:

$$COM_{acc}=\frac{f}{m}$$ (1)

where *f* represents the total force in the AP direction, and *m* represents the body mass.

The amplitude of COM_acc_ variability was quantified using the SD:

$$SD=\sqrt{\frac{1}{n}\sum_{i=1}^{n}{\left(x(i)-\bar{x}\right)}^{2}}$$ (2)

where *x*(*i*) is the magnitude of the COM_acc_ signal at each time point *i*, $\bar{x}$ is the mean value of the *x*(*i*), and *n* is the signal length of *x*(*i*).

The sEMG signals were filtered by a 10–500 Hz band-pass filter and 50 Hz with its odd harmonics notch filter. The amplitude variability of sEMG was estimated by the RMS:

$$RMS=\sqrt{\frac{1}{n}\sum_{i=1}^{n}\text{e}{\left(i\right)}^{2}}$$ (3)

where *e*(*i*) is the amplitude of the sEMG signal at each time point *i*, and *n* is the signal length of *e*(*i*).

**Fig 2:**
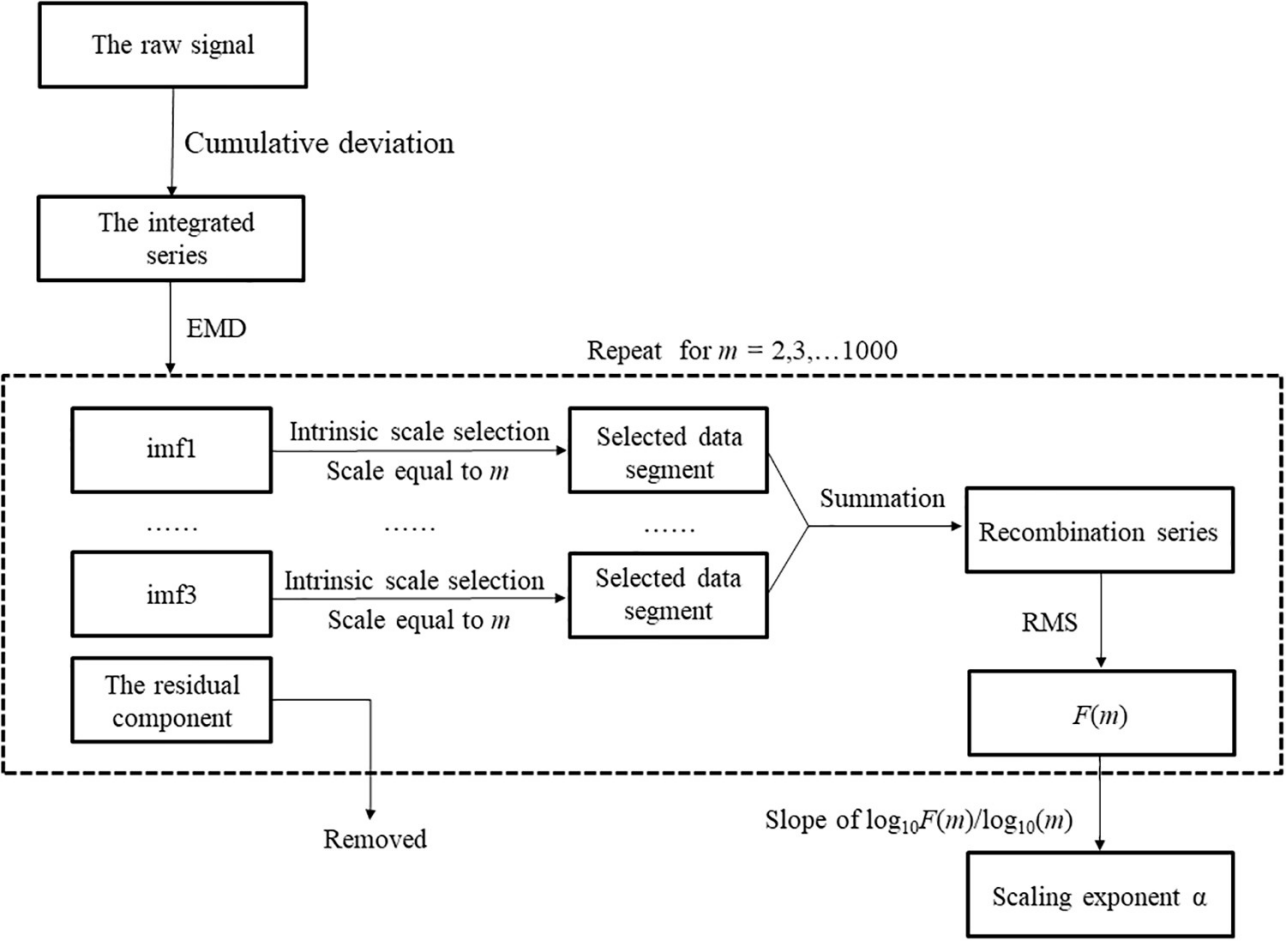
The flow chart of the modified detrended fluctuation analysis with the empirical mode decomposition (EMD-DFA).

**Fig 3:**
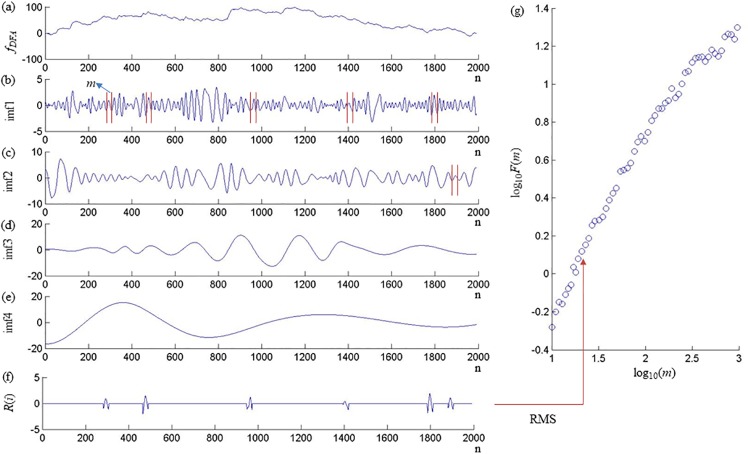
Processing of a representative signal using the EMD-DFA.

The structural variability of sEMG and COM_acc_ signals were estimated using the EMD-DFA algorithm. The flow chart of the algorithm is illustrated in Fig 2. First, the raw COM_acc_ and sEMG signals *x*(*i*) were calculated and integrated as the cumulative deviation:

$$f_{DFA}=\sum_{i=1}^{k}\left(x(i)-\bar{x}\right)$$ (4)

where $\bar{x}$ is the mean of the original time series, and *k* = 1,2,…*n*. Second, the integrated signal *f*_*DFA*_ was decomposed into a set of intrinsic mode functions (imfs) and a residual component following the EMD algorithm (Fig 3) [29]. A set of residual component representing a nonstationary trend of the *f*_*DFA*_ was removed from the following analysis.

In each imf, the intrinsic scales were identified by extracting the data points between each neighboring local minima within windows of equal length *m* (*m* = 2–1000) from all the imfs. All the selected intrinsic scales from all the imfs were resampled such that the zeros were substituted for all data points, and reconstructed as a new time-series *R*(*m*). *F*(*m*) was then estimated as the RMS of *R*(*m*) for all the points (*i* = 1, 2,…*N*).

$$F(m)=\sqrt{\frac{1}{N}\sum_{i=1}^{N}R{\left(m,i\right)}^{2}}$$ (5)

where the *N* was the length of recombination series *R(m*). The α was the slope of the log_10_(m) versus log_10_F (m) in the plot with alteration of window size from 10 to 100. This window size was corresponding to the spectrum of the sEMG signals that mainly ranges between 10 Hz and 100 Hz. All the signals were processed using MATLAB 2014a (The Mathworks, Natick, MA, USA).

Statistical analyses were performed using SPSS 23.0 (SPSS Inc., Chicago, IL). A two-way repeated measures ANOVA was performed to evaluate the effects of group (post-stroke patients vs. healthy subjects) and vision (VF vs. NVF) on the RMS and α of sEMG, as well as the SD and α of COM_acc_. An independent *t*-test was used to examine the differences of sEMG parameters between the paretic limbs and their controls, and between the non-paretic limbs and their controls. In addition, an independent *t*-test was used to examine the differences of the COM_acc_ between the patients and healthy subjects under the two visual conditions. A paired *t*-test was performed to compare the COM_acc_ between the two visual conditions, and to compare the sEMG parameters between the paretic and non-paretic limbs of the patients. Correlation analyses were performed between the sEMG parameters (RMS and α) of TA and LG and the COM_acc_ on the paretic and non-paretic sides of patients and the controls. A *p*-value of less than 0.05 was used to determine statistical significance.

## Results

**Fig 4:**
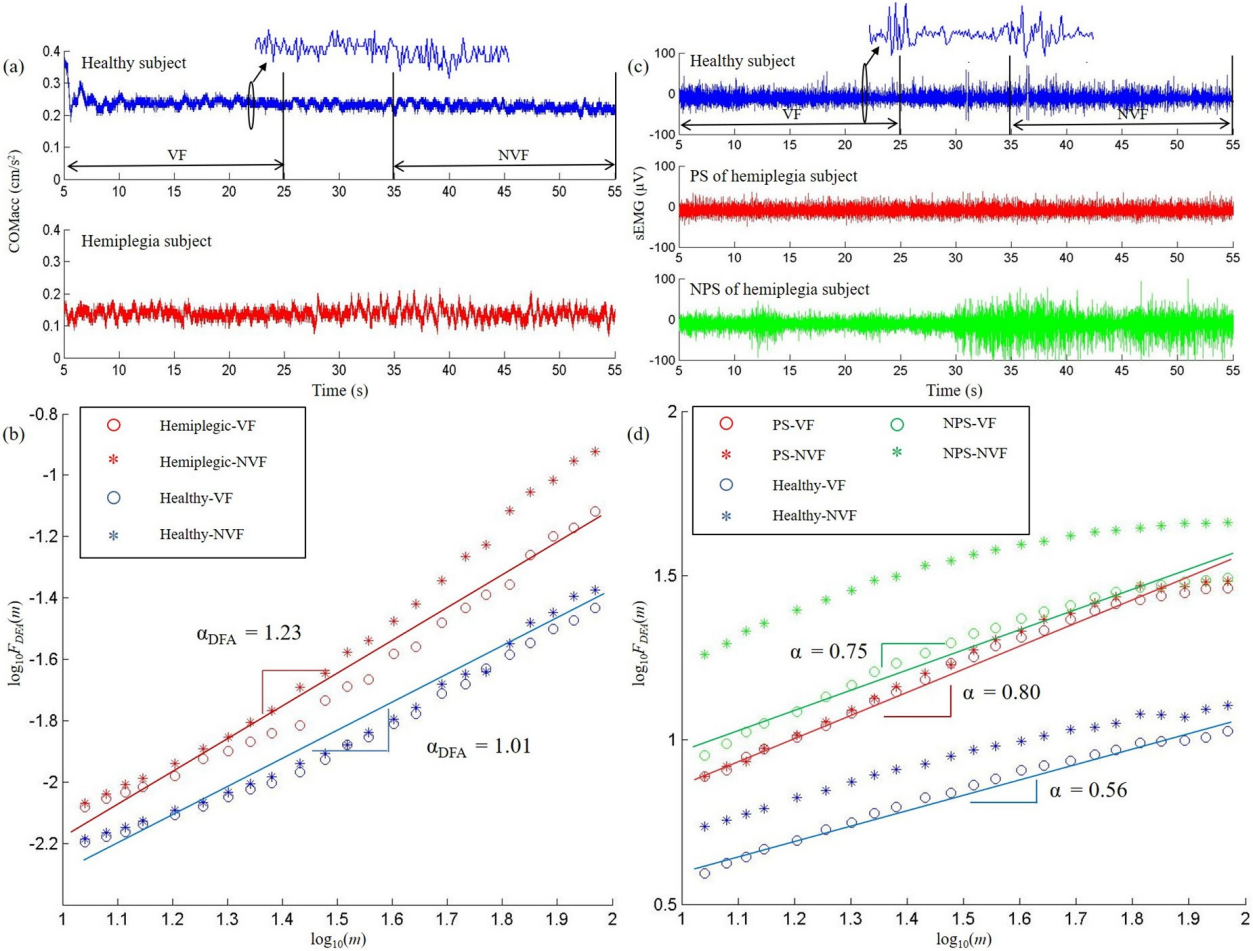
Representative signals and their scaling exponent calculated using the EMD-DFA. (a) The center of mass acceleration (COM_acc_) in anteroposterior (AP) direction from a representative hemiplegic subject and a healthy subject, (b) the EMD-DFA scaling exponents of the COM_acc_ shown in (a) on a log-log plot; (c) the sEMG signals of lateral gastrocnemius (LG) from a representative hemiplegic subject and a healthy subject, (d) the EMD-DFA scaling exponents of the sEMG of LG shown in (c) on a log-log plot.

Fig 4 shows an example of the COM_acc_ (Fig 4(a)) and sEMG (Fig 4(c)) signals of the LG muscle from a representative hemiplegic subject and a healthy subject. The EMD-DFA index of the representative signals were depicted in log-log plots (Fig 4(b) and 4(d)). The hemiplegic subject showed greater fluctuations in the COM_acc_ signals than the healthy subject (Fig 4(a)). In addition, the hemiplegic subject showed decreased sEMG amplitudes on paretic limbs but increased sEMG amplitudes on non-paretic limbs than the control limbs of healthy subjects especially under the NVF condition (Fig 4(c)). Both the COM_acc_ and sEMG signals showed positive EMD-DFA exponents in the log-log plots (Fig 4(b) and 4(d)). In addition, the hemiplegic subject showed higher scaling exponents (α) of COM_acc_ and sEMG according the EMD-DFA algorithm than the healthy subject.

**Fig 5:**
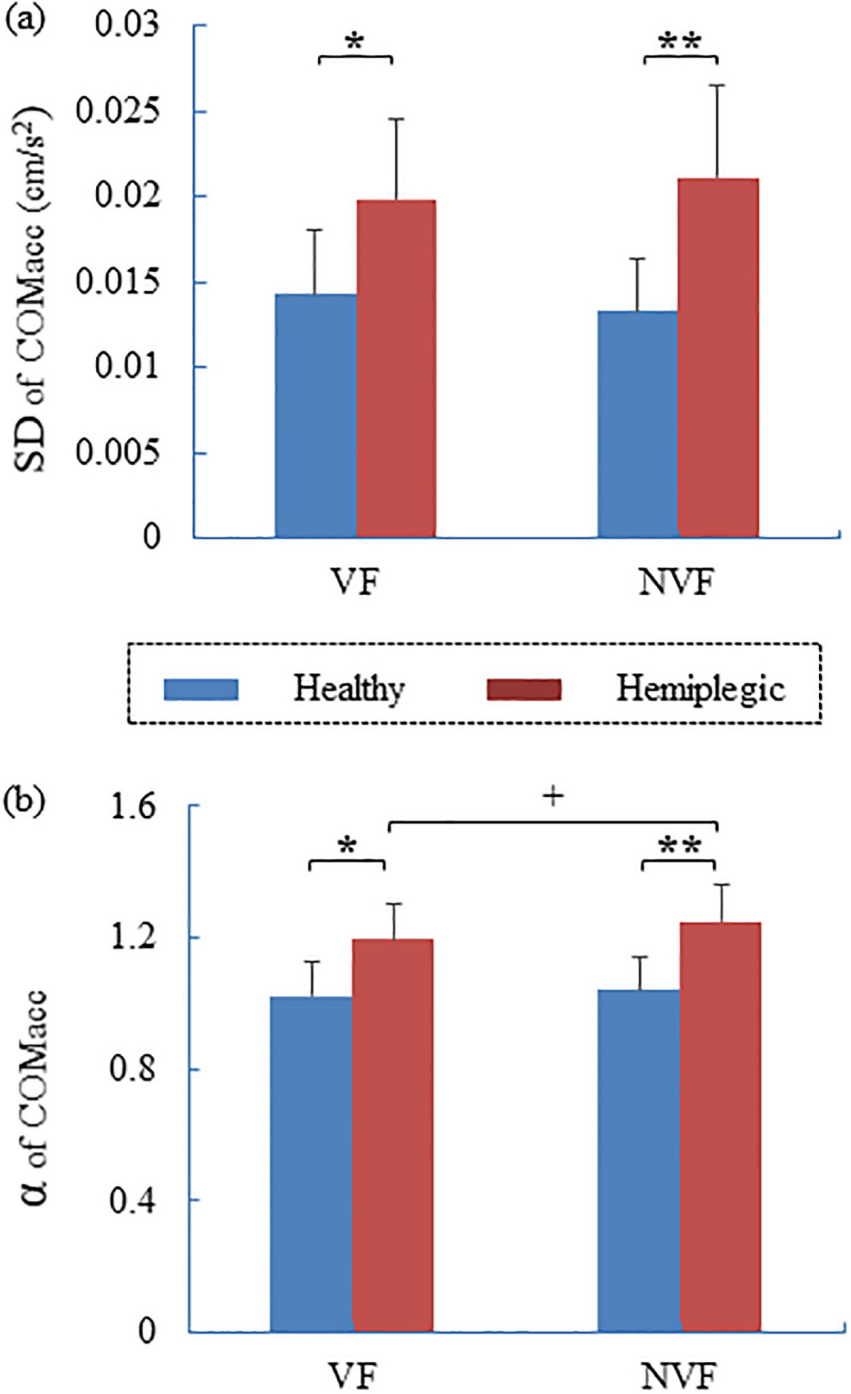
The COM_acc_ in hemiplegic and healthy groups. (a) The standard deviation (SD) and (b) scaling exponent (α) of EMD-DFA. * *p* < 0.05, ** *p* < 0.001. ^+^ Significant difference between the two visual conditions (^+^*p* < 0.05).

Results of statistical comparisons between the hemiplegic and control limbs are presented in Table 2. Repeated measures ANOVA showed that the SD (*F*_1, 20_ = 15.464, *p* < 0.001, Fig 5(a)) and α (*F*_1, 20_ = 18.687, *p* < 0.001, Fig 5(b)) of the COM_acc_ of patients were significantly higher than those of healthy subjects under both VF (SD: *t* = -3.050, *p* < 0.01; α: *t* = -3.825, *p* < 0.01) and NVF (SD: *t* = -4.136, *p* < 0.001; α: *t* = -4.532, *p* < 0.001) conditions (Table 2). Significant differences between the VF and NVF conditions were observed in the α of COM_acc_ (*F*_1, 20_ = 8.950, *p* < 0.01, Fig 5(b)). Without visual feedback, the α values of COM_acc_ were significantly higher than the values with visual feedback in stroke patients (*t* = -3.883, *p* < 0.01, Fig 5(b)).

**Table 2:** Statistical analysis for amplitude and structural variability of patients and controls.

Variability	Parameters	Groups	Visual	Non-Visual
Amplitude Variability	SD of COM_acc_	Hemiplegic	0.020 ± 0.004*	0.021 ± 0.005**
		Healthy	0.014 ± 0.004	0.013 ± 0.003
	RMS of TA	Paretic	14.182 ± 2.138	14.790 ± 2.278
		Non-Paretic	18.712 ± 4.081*	21.151 ± 5.927*
		Healthy	13.678 ± 2.670	13.554 ± 2.139
	RMS of LG	Paretic	18.649 ± 6.960	18.473 ± 7.483
		Non-Paretic	23.934 ± 7.735*	24.878 ± 8.502*
		Healthy	16.030 ± 2.616	17.082 ± 3.186
Structural Variability	α of COM_acc_	Hemiplegic	1.196 ± 0.101*	1.249±0.106**
		Healthy	1.023 ± 0.102	1.040 ± 0.100
	α of TA	Paretic	0.678 ± 0.294*	0.757 ± 0.251*
		Non-Paretic	0.629 ± 0.190*	0.770 ± 0.181**
		Healthy	0.422 ± 0.211	0.494 ± 0.206
	α of LG	Paretic	0.347 ± 0.214	0.421 ± 0.259
		Non-Paretic	0.374 ± 0.322	0.488 ± 0.300
		Healthy	0.304 ± 0.118	0.333 ± 0.165

Data are presented as mean±standard deviation.

* Significant differences from the healthy subjects, *p* < 0.05.

** Significant differences from the healthy subjects, *p* < 0.001.

**Fig 6:**
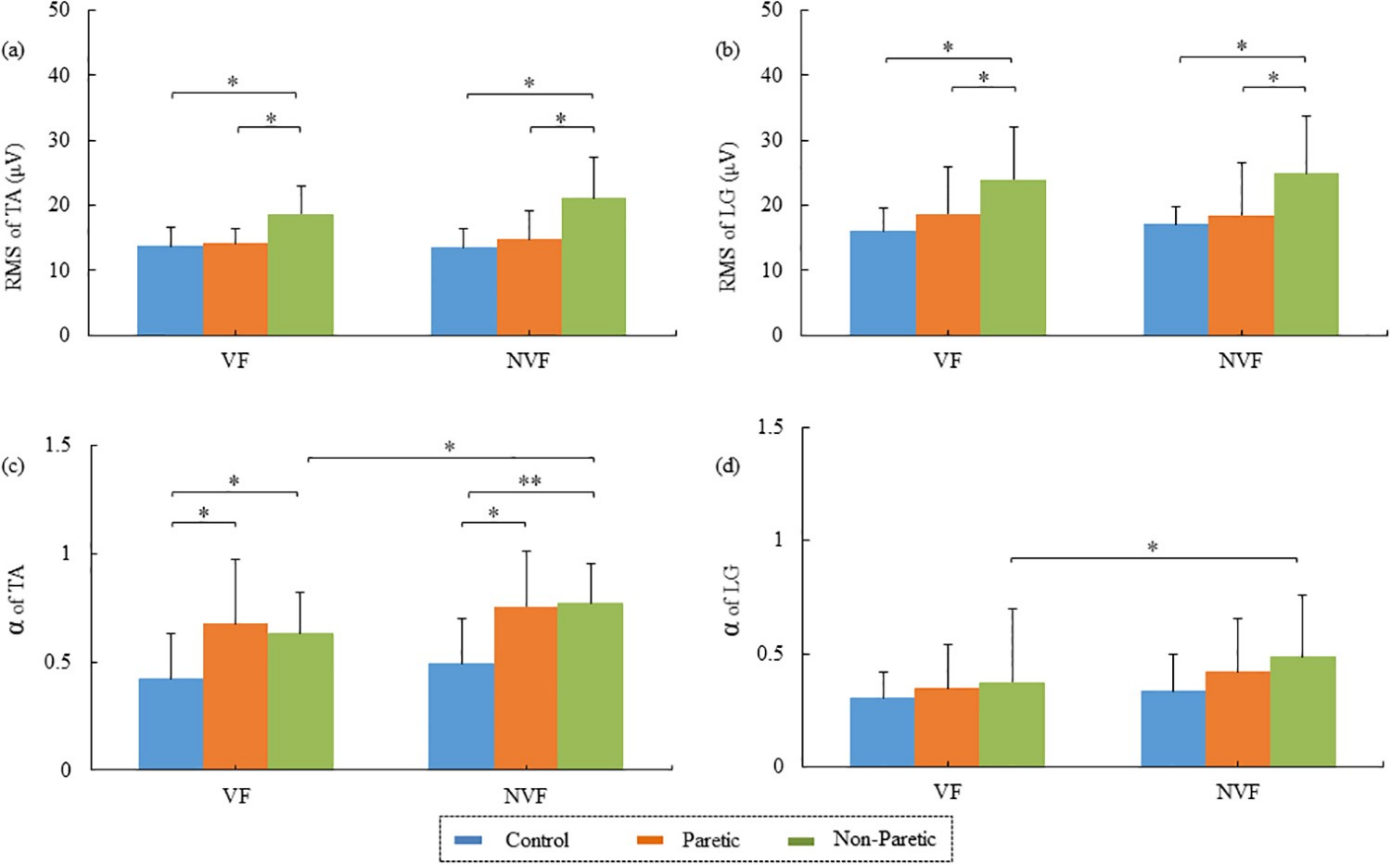
Muscle activations in the hemiplegic and healthy groups. (a) The root mean square (RMS) of tibialis anterior (TA); (b) RMS of LG; (c) scaling exponent (α) of TA; and (d) α of LG. * *p* < 0.05, ** *p* < 0.001).

The repeated measures ANOVA further showed main effects of hemiplegic paralysis on the RMS of TA (*F*_2, 20_ = 13.853, *p* < 0.001, Fig 6(a)) and LG (*F*_2, 20_ = 4.086, *p* < 0.05, Fig 6(b)). No significant difference was observed between the VF and NVF for either the RMS of TA (*p* = 0.180, Fig 6(a)) or the RMS of LG (*p* = 0.193, Fig 6(b)). Specifically, the RMSs of TA on non-paretic limbs were significantly higher than those on paretic limbs (VF: *t* = -2.896, *p* < 0.05; NVF: *t* = -2.809, *p* < 0.05) in the patients. In addition, the RMSs of TA on non-paretic limbs in the patients were significantly higher than those of the control limbs in healthy subjects (VF: *t* = -3.253, *p* < 0.01; NVF: *t* = -3.813, *p* < 0.01, Fig 6(a)). No significant difference was observed in the RMS of TA between the paretic limbs of patients and the control limbs of healthy subjects (VF: *p* = 0.649; NVF: *p* = 0.226, Fig 6(a)). For the RMS of LG, the non-paretic limbs of patients were significantly higher than their paretic limbs (VF: *t* = -3.678, *p* < 0.01; NVF: *t* = 2.873, *p* < 0.05) as well as the control limbs of healthy subjects (VF: *t* = -3.016, *p* < 0.01; NVF: *t* = -2.715, *p* < 0.05, Fig 6(b)). No significant difference was observed in the RMS of LG between the paretic limbs of post-stroke patients and the control limbs of healthy individuals (VF: *p* = 0.286; NVF: *p* = 0.597, Fig 6(b)).

Results of EMD-DFA analysis further showed main effects of hemiplegic paralysis on α of TA (*F*_2, 20_ = 9.170, *p* < 0.01, Fig 6(c)) and LG (*F*_2, 20_ = 4.869, *p* < 0.05, Fig 6(d)). Both the paretic (VF: *t* = -2.241, *p* < 0.05; NVF: *t* = -2.562, *p* < 0.05) and non-paretic limbs (VF: *t* = -2.312, *p* < 0.05; NVF: *t* = -3.187, *p* < 0.005, Fig 6(c)) of patients showed augmented α of TA than the control limbs of the healthy individuals. No significant difference was found in α values of TA between the paretic and non-paretic limbs of patients (VF: *p* = 0.551; NVF: *p* = 0.850, Fig 6(c)). By contrast, no significant difference was observed in α of LG among the paretic, non-paretic and control limbs under both VF (paretic vs. control: *p* = 0.305, non-paretic vs. control: *p* = 0.530, paretic vs. non-paretic: *p* = 0.961) and NVF (paretic vs. control: *p* = 0.184, non-paretic vs. control: *p* = 0.069, paretic vs. non-paretic: *p* = 0.525, Fig 6(d)) conditions. Visual feedback affected the α of TA (*F*_2, 20_ = 5.184, *p* < 0.05, Fig 6(c)) and LG (*F*_2, 20_ = 8.376, *p* < 0.01, Fig 6(d)). In stroke patients, the α of TA and LG of non-paretic limbs significantly increased after removing visual feedback (TA: *t* = -2.713, *p* < 0.05, Fig 6(c), LG: *t* = -3.258, *p* < 0.01, Fig 6(d)). No significant effects of visual feedback on α of TA and LG of paretic limbs (TA: *p* = 0.441, Fig 6(c), LG: *p* = 0.170, Fig 6(d)) or on α of the control limbs (TA: *p* = 0.143, Fig 6(c), LG: *p* = 0.339, Fig 6(d)).

The Table 3 exhibits correlations between the muscle activities and the COM_acc_ on the paretic, non-paretic and control groups. The SD of COM_acc_ was positively correlated with the RMSs of TA under VF (*r* = 0.624, *p* < 0.05, Table 3); and the α of COM_acc_ was positively correlated with the α of LG under NVF (*r* = 0.619, *p* < 0.05, Table 3), on the non-paretic side of the patients. Neither for the paretic group nor for the control group had significant correlations between the COM_acc_ and the muscles activations (Table 3).

**Table 3:** Correlations between the COM_acc_ and the sEMG variabilities.

		Paretic		Non-paretic		Healthy	
**COM**_**acc**_**_SD**	**Condition**	**TA_RMS**	**LG_RMS**	**TA_RMS**	**LG_RMS**	**TA_RMS**	**LG_RMS**
	**VF**	*r* = 0.171	*r* = -0.238	***r* = 0.624**	*r* = -0.113	*r* = -0.484	*r* = 0.520
		*p* = 0.614	*p* = 0.480	***p* < 0.05**	*p* = 0.741	*p* = 0.132	*p* = 0.101
	**NVF**	*r* = 0.308	*r* = -0.388	*r* = -0.128	*r* = -0.054	*r* = -0.035	*r* = 0.313
		*p* = 0.356	*p* = 0.238	*p* = 0.707	*p* = 0.875	*p* = 0.918	*p* = 0.348
**COM**_**acc**_**_α**	**Condition**	**TA_α**	**LG_α**	**TA_α**	**LG_α**	**TA_α**	**LG_α**
	**VF**	*r* = -0.343	*r* = 0.249	*r* = 0.194	*r* = 0.506	*r* = 0.297	*r* = 0.179
		*p* = 0.302	*p* = 0.461	*p* = 0.567	*p* = 0.112	*p* = 0.376	*p* = 0.598
	**NVF**	*r* = 0.451	*r* = 0.552	*r* = 0.362	***r* = 0.619**	*r* = 0.548	*r* = 0.310
		*p* = 0.164	*p* = 0.078	*p* = 0.274	***p* < 0.05**	*p* = 0.081	*p* = 0.353

## Discussion

This study investigated the effects of stroke-induced hemiplegic paralysis on postural stability during quiet standing. The transition during stepping on or stepping off the force plate contains information more relevant to the dynamic rather than static equilibrium; and were thus excluded from the analysis. Results showed increased SD and α of COM_acc_ in hemiplegic paralysis. The COM_acc_ is considered to be an indicator of the synchronization between the COM and COP of the postural control system [30]. The increased amplitude variability of COM_acc_ quantified by SD in the AP direction suggests reductions in the COP-COM synchronization and postural stability in hemiplegic individuals after stroke [6]. It is noteworthy that the effects of hemiplegic paralysis on the COM_acc_ were more evidently observed under the NVF rather than the VF condition, indicating deteriorated COP-COM synchronization and growing instability without visual information. This finding was in line with the previous study showing that the hemiplegic patients relied more on visual feedback for keeping postural stability during standing [31].

Previous studies found that the α ≈ 1 indicates a “pink noise” pattern of the signals’ structure, revealing robust control strategy and high dynamic stability of a complex system [24, 26]. The α between 1 and 1.5 indicates a transition from “pink noise” to “Brownian noise”, which reflects a compromised flexibility in the control strategy and reduced dynamic stability of a complex system [32–34]. The current study found that the α values of COM_acc_ were approximately 1.2 for the patients and 1 for the healthy subjects, suggesting a decrease in the flexibility of posture control in hemiplegic paralysis. This decreased structural variability of COM_acc_ may be attributed to functional degradation in neuromuscular system [35, 36], or deficits in integrating sensorimotor information for maintaining balance [37].

Asymmetries of muscle contraction variability during standing were also observed between the paretic and non-paretic limbs in stroke patients. The TA and LG of the paretic limbs had higher amplitude variability than those of the non-paretic limbs, which may suggest a higher contribution of the non-paretic muscles in maintaining standing balance than the paretic side in hemiplegic paralysis [38]. No significant difference was found in the amplitude variability of muscle contractions between the paretic limbs of the stroke patients and the control limbs of healthy subjects, which was consistent with the previous finding that the paretic limbs of stroke patients had comparable amplitude variability of muscle contraction to the limbs of healthy individuals [16].

The α values of sEMG signals of the TA and LG on the healthy limbs of the controls were between 0 and 0.5, which is indicative of a low negative self-correlation and anti-persistence of neuromuscular control for maintaining stable standing posture [36]. Previous studies have found that hemiplegic individuals had reduced coordination complexity of muscle activation in lower extremities for posture control during locomotion [39]. Considering the TA was responsible for the sway in AP direction during standing, the increased α (between 0.5 and 1) of TA on paretic and non-paretic limbs of hemiplegia subjects versus on the control limbs of the healthy subjects revealed moderate positive self-correlation and a loss of anti-persistence characteristic of neuromuscular activation for keeping balance at the AP direction during quiet standing. This could be attributed to a chronic fatigue or degradation of nervous system in the lower limbs due to stroke [40, 41]. The TA and LG showed different structural variabilities, which may be related to the different roles of the two muscles in standing balance control [37, 42]. In addition, the α values of TA and LG on non-paretic rather than paretic limbs augmented after removing the feedback, which may further substantiate that without visual supervision the postural control would be more reliant on the non-paretic rather than the paretic limbs in the patients with hemiplegia [31].

**Fig 7:**
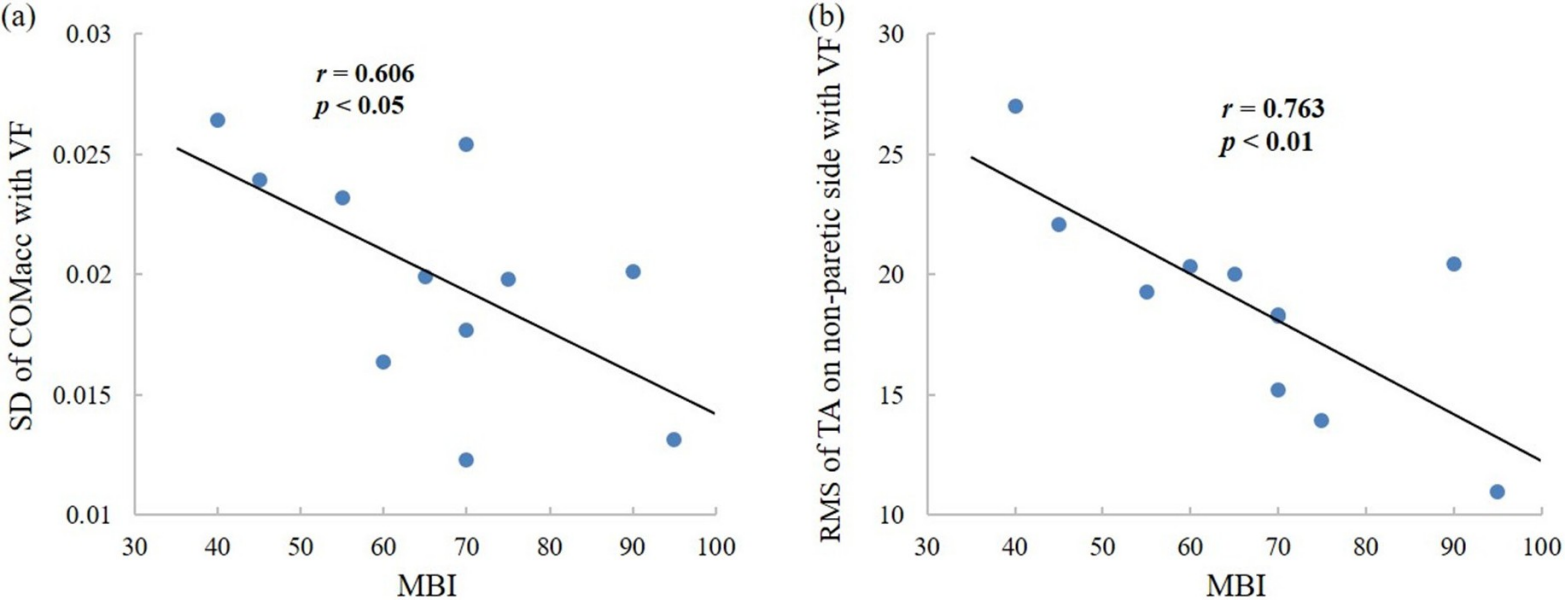
Correlations in the hemiplegic individuals between the Modified Barthel Index (MBI) and (A) the SD of COM_acc_ with visual feedback; (B) the RMS of TA on non-paretic side with visual feedback.

This study investigated the amplitude and structural variabilities of COM_acc_ and sEMG within the same framework for post-stroke patients. Positive correlations were found between the COM_acc_ and the sEMG variabilities for the non-paretic limbs, suggesting the strengthened relationship between the postural variability and the responsible muscle activations in the non-paretic limb of post-stroke patients. No significant correlations were found between the EMG and COM_acc_ for the other parameters, which suggest the relationship between the muscles and the COM_acc_ may not fit linear correlations. Considering the TA and LG are only two representative muscles related to the posture stability but not all, more muscles may also contribute to the balance control. Advanced biomechanical modelling, inverse kinematics or software packages such as OpenSim would be more suitable to find the relationship between specific muscles and the COM. In order to examine whether the COM_acc_ and the muscle activations could reflect the neuropathological characteristics of the patients, correlation analysis were further performed for the COM_acc_ and sEMG parameters with MBI—an index of capacity of daily activity including balance and posture control (Fig 7). Results showed significant correlations between the MBI and the variabilities of COM_acc_ and as well as of sEMG (Fig 7). These results are in line with the previous findings, which showed that as a variable reflecting the synchronization between the COP and COM, the COM_acc_ could serve as an indicator of balance dysfunction, falling risk or capacity of motor control that may play a role in supervision of post-stroke rehabilitation [6, 9, 35]. For the clinicians, the amplitude and structural variability of COM_acc_ may provide quantified estimate of the deficits in the balance control for the patients with hemiplegic paralysis, and can be used as an independent predictor of the risk of falling. Compared with scales, angular momentums or movement kinematics, the COM_acc_ is objective, quantitative and easy to be recorded, reflecting the interaction and coordination among the joints of whole-body [4, 5]. Analysis of the COM_acc_ and sEMG simultaneously may facilitate to find out the muscles’ contributions to the postural stability, and may improve the standing balance by training the specific muscles.

This study may have some limitations. First, the sEMG signals were recorded only from the TA and LG—two representative muscles with major contribution to the COM control particularly in the AP direction. More muscles, such as musculus glutaeus maximus or the COM_acc_ in the medial-lateral direction are worthy to be examined. Second, the laterality or limb preference would be an interfering factor that potentially influence both the patients and the controls. The current study had not taken into account the limb preference for either the patients or the controls. Third, the limited data of the current study cannot reveal the relationship between the variabilities of COM_acc_ and sEMG and the more behavioral or clinical outcome measures. Further studies are needed to investigate the potential relationship between the COM_acc_ and sEMG variabilities and the postural corrections by different time-scale/mechanisms, motor unit recruitment pattern, torque generation, or the task demand.

## Conclusions

Patients with hemiplegic paralysis demonstrated an increased amplitude variability but decreased structural variability of COM_acc_ during standing, which reflects a reduction in the COP-COM synchronization, deficiency in sensorimotor integration, and loss of flexibility for postural control in stroke patients. The asymmetric amplitude variability between the paretic and non-paretic muscle contractions, as well as the inconsistent structural variability between the TA and LG, may partially explain the changes in COM_acc_ in stroke patients, and further reveal the unequal reliance on the paretic and non-paretic limbs in postural control during standing.
